# A lifespan perspective on depression in the postpartum period in a racially and socioeconomically diverse sample of young mothers

**DOI:** 10.1017/S0033291722001210

**Published:** 2023-07

**Authors:** Alison E. Hipwell, Irene Tung, Robert T. Krafty, Audrey W. Leong, Meredith Spada, Hope Vaccaro, Sarah C. Homitsky, Eydie Moses-Kolko, Kate Keenan

**Affiliations:** 1 Department of Psychiatry, University of Pittsburgh, Pittsburgh, PA, USA; 2 Department of Psychology, University of Pittsburgh, Pittsburgh, PA, USA; 3 Department of Psychology, California State University Dominguez Hills, Carson, CA, USA; 4 Department of Biostatistics and Bioinformatics, Emory University, Atlanta, GA, USA; 5 Department of Biological Sciences, University of Pittsburgh, Pittsburgh, PA, USA; 6 Department of Psychological Sciences, Case Western University, Cleveland, OH, USA; 7 Women's Behavioral Health, Allegheny Health Network, Pittsburgh, PA, USA; 8 Department of Psychiatry and Behavioral Neuroscience, University of Chicago, Chicago, IL, USA

**Keywords:** Cumulative effects, lifespan, Pittsburgh Girls Study, postpartum depression, preconception

## Abstract

**Background:**

Consistent evidence from retrospective reports and case registry studies indicates that a history of depression is a major risk factor for depression in the peripartum period. However, longitudinal studies with racially and socioeconomically diverse samples of young mothers are lacking, and little is known about developmental patterns of depression across the lifespan that can inform preventive interventions.

**Methods:**

Young primiparous mothers (*n* = 399, 13–25 years, 81% Black) were recruited from a population-based prospective study that began in childhood. Women reported on depression symptoms for at least 3 years prior to their pregnancy, during pregnancy, and at 4 months postpartum. Linear regression models were used to estimate change in pre-pregnancy depression severity and to evaluate associations between patterns of lifetime history and postpartum depression symptoms.

**Results:**

Results revealed high levels of continuity in depression from pregnancy to postpartum, and across multiple years pre-pregnancy to postpartum. Overall, depression severity leading up to pregnancy decreased over time, but patterns of worsening or improving symptoms were not associated with depression severity in the postpartum period. Instead, area under the pre-pregnancy trajectory curve, representing cumulative lifetime depression burden, was uniquely associated with postpartum depression after adjusting for prenatal depression severity.

**Conclusions:**

Depression in the postpartum period should be considered within a lifespan perspective of risk that accumulates before conception. Clinical screening and early interventions are needed in adolescence and young adulthood to prevent the onset and persistence of depressive symptoms that could have long-term implications for peripartum health.

Depression occurring in the year following childbirth remains a serious public health concern affecting 10–15% of women in the USA (O'Hara & McCabe, 2013; Shorey et al., 2018). Evidence suggests that the burden of postpartum depression may be especially high among adolescent and young adult women, Black women, and women living in under-resourced environments (Goyal, Gay, & Lee, 2010; Pooler, Perry, & Ghandour, 2013), likely reflecting higher levels of concomitant stress, financial strain, and inequities in mental health access and care. Depression in the postpartum period often goes undetected and untreated (Pearlstein, Howard, Salisbury, & Zlotnick, 2009) and is associated with long-term adverse effects for the mother, child, and her family (Letourneau et al., 2012; Weissman et al., 2006) even when symptoms do not reach diagnostic threshold (Meaney, 2018). These multiple concerns point to an urgent need for the early identification of women who would benefit from targeted, preventive interventions.

Across the lifespan, a history of depression is known to be a strong risk factor for subsequent depression (Rudolph, Flynn, Abaied, Groot, & Thompson, 2009). Around the time of childbirth, short-term prospective studies have shown high levels of continuity in depression from pregnancy to the postpartum period (Davé, Petersen, Sherr, & Nazareth, 2010; Eastwood et al., 2017; Guintivano et al., 2018) that have led to the broader conceptualization of ‘peripartum depression’ encompassing symptoms during and/or after pregnancy (American Psychiatric Association, 2013). There is also consistent evidence that a history of depression at any time *prior* to pregnancy is associated with increased risk for peripartum depression (Davé et al., 2010; Dennis, Heaman, & Vigod, 2012; Guintivano et al., 2018; Räisänen et al., 2014; Silverman et al., 2017). However, the vast majority of these data come from retrospective self-reports, which may be subject to recall bias in ways that are congruent with mood (Eich & Macaulay, 2000; Walker, Skowronski, Gibbons, Vogl, & Thompson, 2003), and which typically lack clinical and temporal specificity (Gjerdingen & Yawn, 2007; Moffitt et al., 2010). In addition, extant research often reflects limited sample diversity in terms of age, race, and income level. Adolescent mothers, for example, are frequently excluded from studies of peripartum depression (e.g. Segre, O'Hara, Arndt, & Stuart, 2007; Smith-Nielsen, Matthey, Lange, & Væver, 2018) and in the USA, health insurance-based registry studies are unlikely to represent all sectors of the population. In some cases, research excludes high-risk women covered on Medicaid (Dietz et al., 2007). Similarly, despite significant racial-ethnic disparities in peripartum outcomes, Black women and other women of color are often under-represented in studies investigating the prevalence of depression around the time of childbirth (Gaynes et al., 2005). The limited representativeness of prior samples has made it difficult to estimate the burden of depression history on peripartum depression, particularly for those who may be most at risk. Furthermore, these and other studies have often relied on binary indicators such as a depression diagnosis, a psychiatric referral, or prescribed medication at an unspecified time (Vliegen, Casalin, & Luyten, 2014). Yet, the severity of depression varies according to age of onset (Hölzel, Härter, Reese, & Kriston, 2011), and typically follows a remitting, episodic course (Kennedy, Abbott, & Paykel, 2004; Mueller et al., 1999) that can itself be prognostic (Brodaty, Luscombe, Peisah, Anstey, & Andrews, 2001). Therefore, developmental patterns of depression across the lifespan may be a critical determinant of peripartum adjustment that could also elucidate optimal timing of screening, treatment planning, and follow-up. Finally, studies have shown that depression in adolescence, even at subthreshold levels, is associated with an increased risk for depression in adulthood (Fergusson, Horwood, Ridder, & Beautrais, 2005; Johnson, Cohen, & Kasen, 2009). Given that the incidence of depression increases dramatically between adolescence and early adulthood, prospective studies that span this age range contribute particularly important information about future depression risk (Pine, Cohen, Brook, Gurley, & Ma, 1998).

Only a few longitudinal studies have examined preconception risk for depression during and after pregnancy. In one of two landmark studies, the 20-year Victorian Intergenerational Health Cohort Study (VIHCS, Spry et al., 2020) followed an adolescent cohort to identify pregnancies among participants when they were 29–36 years old. Among 384 women with 564 pregnancies, Patton et al. (2015) reported that mental health problems (i.e. mixed depression/anxiety) occurring during adolescence and young adulthood conferred an 8.4-fold risk for peripartum depression (assessed across late pregnancy and at 2 and 12 months postpartum) relative to no history. Mental health problems in young adulthood only were associated with a 4.6-fold increased risk, whereas adolescent-limited mental health problems were unrelated to peripartum depression risk. These results were replicated in a second longitudinal cohort study, the 36-year Australian Temperament Project (ATP) Generation 3 (Edwards et al., 2013) in which 393 women reported on 686 pregnancies (Thomson et al., 2021). Subsequent analyses of the VIHCS data also showed that prenatal depression explained only a small part of the association between preconception mental health problems and symptoms of postpartum depression (Spry et al., 2021). Taken together, these findings highlight the importance of pregnancy psychopathology for peripartum depression and suggest that timing (i.e. recency) is an important dimension of risk. However, it is also possible that earlier developing symptoms have cumulative effects that only become evident with increasing age. Some methodological considerations include the focus on mixed depression/anxiety rather than specific depression history, changes in measurement from adolescence to adulthood that raise questions about developmental timing effects, and the inclusion of multiparous women for whom the preconception and prior peripartum periods may coincide.

Two shorter-term prospective studies examined continuity in depression symptoms from preconception to the postpartum period. In a Singapore-based study, participants comprised 18- to 45-year-old women (primarily employed, married, and highly educated) who were planning a pregnancy (Kee et al., 2021). Depression symptoms were measured repeatedly across three 3-monthly intervals prior to conception, during the three trimesters of pregnancy and at 3 months postpartum. Results showed moderate within-person stability in depression severity from preconception to postpartum. However, the limited focus on ‘pregnancy planners’ may have introduced selection bias related to multiple psychosocial variables (e.g. life stress) with relevance for depression, and so the generalizability of the results to the broader population is unclear. Second, a US-based longitudinal study of 147 primiparous adolescent mothers (86% Black) drawn from the current sample examined within-person change in depression severity across two time points: a year prior to pregnancy and within the first 6 months postpartum (Hipwell, Murray, Xiong, Stepp, & Keenan, 2016). Using the same measure at both assessments, the results revealed no overall change in self-reported depression across this period, indicating high levels of continuity. However, this analysis was limited to a single pre-pregnancy assessment and, like the prior study, did not consider the longitudinal course or cumulative burden of depression that is associated with future depression severity in non-childbearing samples (Brodaty et al., 2001).

In summary, convergent evidence has shown substantial continuity in depression severity from pregnancy to postpartum and has also highlighted pre-pregnancy depression as a major risk factor for depression occurring during and after pregnancy. However, rigorous studies that include diverse and representative samples, followed prospectively across the lifespan are lacking, and so little is known about developmental patterns of preconception depression (e.g. worsening symptoms, cumulative effects) that could aid detection of vulnerable individuals and inform the timing of preventive interventions. The current study addresses this gap by leveraging data from a population-based, racially and socioeconomically diverse sample of girls who have completed annual assessments for 20 consecutive years from childhood to young adulthood. Utilizing repeated measures of depression severity across an extended developmental period encompassing preconception, pregnancy, and postpartum provides an unprecedented opportunity to examine change, chronicity, and cumulative effects of depression in first-time mothers, including adolescent mothers. Based on prior work, we hypothesized that there would be continuity in depression from pregnancy to the postpartum period, but that preconception depression would explain unique variance in postpartum depression risk. We also hypothesized that increasing depression symptom severity in the period prior to pregnancy, and a greater lifetime burden or accumulation of preconception depression, would predict higher levels of depression in the postpartum period.

## Method

### Sample

Participants were drawn from the Pittsburgh Girls Study (PGS), an ongoing longitudinal study of 2450 women recruited in childhood (5–8 years old) in 1999–2000. The sample was formed following a city-wide enumeration of 103 238 households in Pittsburgh, PA. Neighborhoods in which at least 25% of the families were living at or below the poverty level were fully enumerated (i.e. all homes were contacted to determine if the household contained an eligible girl), and a random selection of households in all other city neighborhoods were 50% enumerated (Hipwell et al., 2002; Keenan et al., 2010). During the past 20 years, participants have been assessed annually with high rates of retention (89% on average, 86% on completion of wave 19).

Between 2006 and 2017, 679 PGS participants became new mothers. Of this group, 615 participants (90.6%) lived in the Pittsburgh region and were eligible for one of three lab-based postpartum sub-studies. All three sub-studies measured depression using the same validated instrument between 3 and 5 months postpartum to capture the peak period prevalence of depression (Munk-Olsen, Laursen, Pedersen, Mors, & Mortensen, 2006). Depression data from the year of pregnancy, and all years prior to pregnancy, were collected as part of the annual PGS assessments.

The first PGS sub-study conducted between 2006 and 2013 identified 186 primiparous adolescent mothers aged 12–19 years, of whom 147 (79%) completed a research visit on average at 5.27 months (s.d. = 2.02) postpartum. Among the potential participants identified but not enrolled, 36 (19.4%) could not be scheduled, three (1.6%) refused participation and 10 (3.2%) were excluded (infant adopted, infant died, mother living in a residential facility). The second sub-study (2011–2016) included 227 primiparous mothers aged 18–23 years. In this maternal neuroimaging study, 308 PGS participants aged 18 and older were screened for MRI eligibility criteria (e.g. no metal in body) and 43 (14%) were excluded. Of 265 eligible participants, 227 (85.7%) completed a postpartum visit at 4.02 months (s.d. = 1.14). A further 29 (10.9%) women could not be scheduled within the specified window and nine (3.4%) refused participation. Finally, an infant neuroimaging sub-study (2015–2017) included 40 primiparous PGS participants (19–25 years) assessed at 3.16 months (s.d. = 0.61) postpartum. In this study, 121 infants were screened, 20 (13.1%) did not meet eligibility criteria (e.g. low birthweight, <37 weeks gestation at birth), 29 (28.7%) could not be scheduled and 32 mothers (31.7%) refused participation.

The final combined sample for the current analyses comprised 414 first-time mothers (75% of those eligible). Availability of PGS data allowed examination of potential selection bias on two levels. First, we compared those enrolled (*n* = 414) with those eligible but not enrolled (*n* = 138) on sociodemographic and depression severity variables from the PGS wave prior to the pregnancy. These analyses revealed no differences on age, race, receipt of public assistance, or preconception depression severity. Second, we compared those enrolled (*n* = 414) with those not targeted for enrollment (*n* = 265) among all identified first-time mothers in the PGS. Results showed that enrolled participants were younger (*M* = 19.15 years, s.d. = 2.18 *v. M* = 20.16, s.d. = 2.01 respectively, *p* < 0.001) and more likely to identify as Black (84.1% *v.* 74.7% respectively, *p* < 0.01). There were no differences on low-income status or preconception depression severity.

### Procedure

Approval for all study procedures was obtained from the University of Pittsburgh Human Research Protection Office. Written informed consent from the caregiver and verbal assent from the girl were obtained through age 17. From age 18 onwards, all participants provided written informed consent.

### Measures

#### Depression severity in the postpartum period

Participants completed the 10-item Edinburgh Postnatal Depression Scale (EPDS; Cox, Holden, & Sagovsky, 1987) which measures severity of depressed mood, anhedonia, guilt, anxiety, and suicidal ideation experienced in the past week rated on four-point scales (0–3). The EPDS has good psychometric properties (Cox et al., 1987; Murray & Carothers, 1990): a score >12 indicates ‘probable’ depression and a score >10 is within the recommended screening range (National Collaborating Centre for Mental Health, 2007). Internal consistency in the current sample was good (*α* = 0.84).

#### Depression severity prior to childbirth

Self-reported depression symptoms before and during pregnancy were assessed annually using the Adolescent Symptom Inventory (ASI-4; Gadow & Sprafkin, 1998) transitioning to the Adult Symptom Inventory (ASRI-4; Gadow, Sprafkin, & Weiss, 2004) at age 18 years. Participants rated the frequency of DSM-IV symptoms of major depressive disorder plus two related symptoms: low self-esteem and hopelessness. Seven symptoms were rated on four-point scales (0 = never to 3 = very often), and four symptoms (changes in appetite, sleep, activity, concentration) were scored as absent (0.5) or present (2.5) following standard scoring procedures (Gadow et al., 2004). Items were summed to form a total depression severity score in each year. The ASI-4/ASRI-4 depression scale demonstrates convergent and discriminant validity and has been shown to differentiate between clinical and non-clinical samples (Gadow et al., 2004).

#### Covariates

Maternal age at delivery was calculated from date of delivery reported by participants, and confirmed in electronic medical records, relative to date of birth. Participants reported on racial identity as part of the PGS. Low-income status, indicated by receipt of public assistance (0 = none, 1 = assistance from sources such as WIC and Medicaid), was reported by the primary caregiver for PGS participants aged 17 years and younger, and via self-report from age 18 onwards.

### Data analytic plan

Analyses were conducted using *R* v3.6.0 (R Core Team, 2019). For each woman, linear regression models were used to estimate change in depression severity starting from the earliest point prior to and leading up to pregnancy by regressing depression severity onto time from pregnancy. We estimated slopes for each individual and examined the mean slope of change across the full sample. We examined the association between depression in the postpartum period and pattern of depression history in three regression models with the following predictors, respectively: (1) depression during pregnancy; (2) slope of preconception depression trajectory; (3) both depression during pregnancy and slope of preconception depression trajectory. Because area under the curve prior to pregnancy quantifies cumulative history of depression, the coefficient of the slope of depression trajectory in Model (3) is inversely proportional to the cumulative history of depression controlling for depression during pregnancy (see Supplementary material for derivation and illustrative example). The trajectory of depressive symptoms for each woman is a latent quantity that is estimated from her observed data. The accuracy of this estimate for a given woman depends on the number of observations and within-subject variability, which is quantified via the standard error of the estimated slope. For Models (2) and (3), we used the simulation-extrapolation (SIMEX) method to account for the standard errors in estimated slopes and to correct for measurement-error-induced bias in estimates (Carroll, Küchenhoff, Lombard, & Stefanski, 1996). In regression models, errors in predictors such as uncertainty in estimates of latent slopes can lead to invalid inference. The SIMEX approach accounts for these errors to reduce bias. In particular, the approach can account for different standard errors in estimated slopes for different women that is a reflection in part of women having different numbers of observations. All models included age, race, and receipt of public assistance as covariates.

## Results

In the final sample of 414 first-time mothers, 408 (98.6%) completed the EPDS and 399 (96%) contributed at least three time points of data prior to pregnancy (the minimum number needed to estimate depression slopes) and so were included in the present analysis.

Descriptive statistics and bivariate correlations among sociodemographic and study variables are presented in Table 1. Participants ranged in age from 13 to 25 years at the time of delivery (mean = 19.20, s.d. = 2.16). Most women identified as Black (81.2%) or Black and another race (multiracial: 3%) and 15.8% identified as White. During pregnancy, 27.8% of participants lived in a household receiving public assistance. Postpartum depression scores, assessed on average at 4.24 months following delivery, ranged from 0 to 23 (*M* = 4.42, s.d. = 4.68), with 9% of this sample scoring in the clinically significant range (score ⩾12) and 15.9% scoring in the screening range (score ⩾10). Depression in the postpartum period was significantly correlated with history of depression severity across most previous time points (significant up to 11 years prior to pregnancy) and was strongly associated with depression during pregnancy and in the year before pregnancy. None of the sociodemographic variables was correlated with depression in the postpartum period.

**Table 1.** Descriptive statistics and correlations among demographic variables and depression scores across time

	*N*	*M* (s.d.) or *N* (%)	1	2	3	4	5	6	7	8	9	10	11	12	13	14	15	16	17	18
1. Age at delivery	399	19.20 (2.16)	–																	
2. Race^a^	399	336 (84.2%)	−0.18**	–																
3. Public assistance^b^	399	73 (27.8%)	0.03	0.14*	–															
4. Dep postpartum	399	4.42 (4.68)	−0.08	−0.08	−0.02	–														
5. Dep in pregnancy	388	7.08 (5.01)	−0.12*	0.06	0.05	0.32**	–													
6. Dep 1 year pre-preg	385	7.56 (4.91)	−0.08	−0.06	0.04	0.29**	0.46**	–												
7. Dep 2 years pre-preg	378	7.26 (4.76)	−0.14**	0.01	0.07	0.26**	0.41**	0.45**	–											
8. Dep 3 years pre-preg	374	7.55 (4.82)	−0.16**	0.02	0.07	0.22**	0.33**	0.41**	0.55**	–										
9. Dep 4 years pre-preg	371	8.09 (4.78)	−0.17**	−0.02	0.01	0.20**	0.30**	0.27**	0.41**	0.48**	–									
10. Dep 5 years pre-preg	352	8.02 (4.72)	−0.10	−0.14**	0.01	0.20**	0.27**	0.27**	0.40**	0.39**	0.51**	–								
11. Dep 6 years pre-preg	334	8.64 (5.08)	−0.04	0.01	0.08	0.18**	0.32**	0.28**	0.42**	0.37**	0.47**	0.49**	–							
12. Dep 7 years pre-preg	287	8.14 (4.80)	−0.11	0.08	0.04	0.22**	0.25**	0.22**	0.29**	0.27**	0.30**	0.30**	0.53**	–						
13. Dep 8 years pre-preg	236	8.55 (4.99)	−0.07	−0.01	−0.05	0.10	0.04	0.10	0.35**	0.22**	0.22**	0.31**	0.41**	0.49**	–					
14. Dep 9 years pre-preg	177	8.58 (4.18)	−0.11	0.03	−0.08	0.17*	0.19*	0.22**	0.32**	0.14	0.23**	0.27**	0.49**	0.53**	0.52**	–				
15. Dep 10 years pre-preg	109	8.32 (4.42)	−0.08	−0.03	−0.02	0.21*	0.21*	0.30**	0.33**	0.19	0.25*	0.27**	0.36**	0.36**	0.24*	0.53**	–			
16. Dep 11 years pre-preg	60	8.19 (3.67)	−0.04	0.18	−0.04	0.29*	0.18	0.16	0.37**	0.17	0.29*	0.20	0.48**	0.49**	0.39**	0.42**	0.47**	–		
17. Dep 12 years pre-preg	21	9.38 (2.97)	0.05	−0.25	0.18	0.41	0.35	0.20	0.31	0.28	−0.22	0.12	0.06	0.29	0.45*	0.19	0.15	0.53*	–	
18. Dep 13 years pre-preg	8	7.08 (5.01)	0.37	−0.22	−0.20	0.02	−0.12	−0.14	0.10	0.02	0.05	−0.11	0.47	0.58	0.32	0.41	0.35	0.07	0.04	–

Dep, depression severity; pre-preg, pre-pregnancy.

a Race coded as 1 = Black American (includes multiracial Black and another race) *v*. 0 = White American.

b Public assistance coded as 0 = no *v*. 1 = yes. Postpartum depression was measured by the Edinburgh Postnatal Depression Scale, whereas depression during pregnancy and all pre-pregnancy time points was measured by the Adolescent/Adult Self-Report Inventory-4 major depressive disorder scale. All available pre-pregnancy time points were used; thus, depending on age of childbirth, some participants had more years of depression data available before pregnancy than other participants.

*p < 0.05, **p < 0.01.

Overall, there was a significant decrease in depression severity over time prior to childbirth, with an estimated mean slope of *B* = −0.20, *p* < 0.01 (95% CI −0.27 to −0.12). However, there was substantial variability in slopes (stable, increasing, decreasing) across individuals, with negative and positive slope gradients ranging from *B* = −3.10 to 3.20 respectively.

### Predicting depression severity in the postpartum period

Results from the regression models predicting postpartum depression severity appear in Table 2. Adjusting for age at delivery, race, and low-income status, depression severity during pregnancy significantly predicted depression in the postpartum period (Model 1): a three-point increase in prenatal depression severity was associated with more than a one-point increase in severity of postpartum depression (*B* = 1.17). Without considering individual differences in depression severity during pregnancy, the slope of the preconception depression trajectory was not associated with depression in the postpartum period (Model 2). When Model 2 was re-run without adjusting for age at childbirth or receipt of public assistance, the results were unchanged, suggesting that these intermediary variables were not operating as mediators. When simultaneously considering the association of depression during pregnancy and the slope of preconception depression on depression in the postpartum period (Model 3), prenatal depression remained positively associated with depression in the postpartum period (*B* = 0.52, s.e. = 0.08, *p* < 0.001), whereas the slope of preconception depression trajectory was negatively associated with postpartum depression severity (*B* = −1.44, s.e. = 0.55, *p* < 0.01). After adjusting for differences in depression during pregnancy, a negative depression trajectory slope reflects greater levels of cumulative depression leading up to pregnancy (i.e. area under the curve of preconception depression trajectory). Consequently, these results suggest that beyond continuity in depression from pregnancy to postpartum, the cumulative history of depression explains additional unique variance in depression in the postpartum period such that an average three-point increase in depression severity per year over 8 years (the median preconception observation time in our sample) is associated with an estimated 1.08 increase in expected EPDS score (s.e. = 0.41, *p* < 0.01). To explore the possible effects of missingness, we refit the model using only data from women with complete data. In a sensitivity analysis, we compared inference from the reported model to this complete case model and found point estimates to be highly similar.

**Table 2.** Regression models predicting postpartum depression scores from history of depression

	*B*	s.e.	Partial *R*^2^	*p* Value
Model 1				
Age at delivery	−0.11	0.11	0.004	0.332
Race^a^	−1.79	0.75	0.021	0.018
Prenatal public assistance^b^	−0.18	0.62	0.050	0.769
Depression severity during pregnancy	0.39	0.06	0.139	<0.001
Model 2				
Age at delivery	−0.16	0.12	0.008	0.175
Race^a^	−1.54	0.81	0.014	0.058
Prenatal public assistance^b^	0.02	0.66	0.002	0.976
Slope of preconception depression	0.48	0.49	0.003	0.328
Model 3				
Age at delivery	−0.15	0.11	0.005	0.183
Race^a^	−1.88	0.75	0.023	0.012
Prenatal public assistance^b^	−0.32	0.61	0.063	0.600
Depression severity during pregnancy	0.52	0.08	0.157	<0.001
Slope of preconception depression	−1.44	0.55	0.024	0.009

*Note*. Models are adjusted for race, prenatal public assistance, and slope of preconception depression. Significant effects are bolded for emphasis.

a Race coded as 1 = Black American (includes multiracial Black and another race) *v*. 0 = White American.

b Public assistance coded as 0 = no *v*. 1 = yes.

Regarding the role of demographic factors in the model, neither age at delivery nor public assistance predicted depression in the postpartum period after accounting for history of depression. Furthermore, adjusting for these variables did not significantly reduce estimates of depression continuities in Model 2. However, there was a main effect of race on depression in the postpartum period, with Black mothers reporting lower levels of depression than White mothers after adjusting for age, public assistance, depression during pregnancy, and slope of preconception depression trajectory (Table 2). Given the significant negative association between Black race and depression in the postpartum period, we further probed the nature of this association with respect to history of depression prior to childbirth. Black participants did not differ from White participants on depression severity reported during pregnancy (*t* = 1.10, *p* = 0.27) or in terms of slope of preconception depression trajectory (*t* = 0.04, *p* = 0.97).

## Discussion

The current study provides evidence that depression in the postpartum period should be considered in the context of depression that has *accumulated* across the life course prior to pregnancy. These findings emerged from a rigorous longitudinal study of racially and economically under-represented, young first-time mothers who had been followed annually since childhood. Approximately one in six of the young women reported postpartum depressive symptoms within the recommended screening range (National Collaborating Centre for Mental Health, 2007); a rate that is largely consistent with other community-based samples.

The study builds on existing literature in several critical ways. First, the current analyses utilized multiple waves of repeated-measures data to reveal high levels of continuity, not just from pregnancy to postpartum, but also from an extended period before pregnancy to the early months postpartum. Not surprisingly, depression severity in the years closest to childbirth was most strongly associated with depression in the postpartum period. However, postpartum depression severity was also correlated with symptom scores across multiple years prior to pregnancy commensurate with the chronic nature of depression symptomatology in adolescents and young adults (Wesselhoeft, Sørensen, Heiervang, & Bilenberg, 2013) and highlighting the importance of the preconception life stage, specifically the adolescent period, for capturing postpartum vulnerability in first-time mothers. These results are similar to those reported in two Australian cohort studies showing that mental health problems (i.e. mixed depression/anxiety) during adolescence and adulthood elevate risk for depression in the postpartum period (Patton et al., 2015; Spry et al., 2020; Thomson et al., 2021). However, here we provide evidence for both short- and longer-term continuities that are specific to depression and were observed in an urban US sample of young, predominantly Black women.

Second, the results showed that on average, depression severity leading up to childbirth decreased over time in this young mother sample. This finding is reminiscent of declining rates of depression across the peripartum period (Banti et al., 2011; Dipietro, Costigan, & Sipsma, 2008; Patton et al., 2015; Thomson et al., 2021), which increased the clinical and research focus on pregnancy as a key window of risk. However, in the current study, patterns of developmental change in preconception depression severity (whether increasing, decreasing, or stable) were unrelated to depression severity in the postpartum period. This has important implications for clinical translation suggesting that knowledge of symptom severity change *per se* may have limited utility in the detection of vulnerable postpartum women.

Third, the current study highlighted cumulative burden of depression across the lifespan as an important risk factor for depression in the postpartum period. Specifically, burden operationalized as area under the preconception trajectory curve was uniquely associated with depression in the postpartum period after adjusting for severity of prenatal depression. This finding is consistent with life-course theories describing accumulating and cascading effects of early adverse influences on future mental and physical health (Cicchetti & Toth, 2009; Geronimus, Hicken, Keene, & Bound, 2006; Gutman, Joshi, & Schoon, 2019). The accumulation of early-onset, episodic, and/or enduring depression could elevate depression vulnerability via multiple pathways including alterations in biological stress response systems (Hamilton & Alloy, 2016; Liu, 2017), maladaptive cognitive styles (Monroe & Harkness, 2005), poor coping skills (Cairns, Yap, Pilkington, & Jorm, 2014), and impaired interpersonal relationships (Shih, Barstead, & Dianno, 2018); each of which has also been linked to depression in the postpartum period (Glynn, Davis, & Sandman, 2013; Gotlib, Whiffen, Wallace, & Mount, 1991; Hipwell et al., 2004; Howard, Oram, Galley, Trevillion, & Feder, 2013). Gaining an understanding of the ways in which lifetime burden of depression heightens risk for depression in the postpartum period via altered biology and modifiable risk factors is an important avenue for future investigation.

The current study used a person-centered approach to examine individual trajectories of change in depression severity and their associations with depression in the postpartum period. Although the model accounted for variability in the course of preconception depression showing stable, increasing, or decreasing patterns, there may have been meaningful classes of individuals with distinct longitudinal trajectories (Guyon-Harris, Huth-Bocks, Lauterbach, & Janisse, 2016) that were not accounted for. Thus, for some women, depression in the postpartum period may be biologically and phenotypically distinct from depression occurring at other times in their life, and for others depression may be experienced *de novo* in the postpartum period. It is therefore possible that the results reported here underestimate the strength of the cumulative burden of lifetime depression for women with a prior history.

Finally, Black women reported lower levels of depression in the postpartum period than White women in our sample, but there were no differences in prenatal depression severity or the pre-pregnancy depression slope. However, the proportionally small sample of White women (16%) precluded any inferences being drawn about differential patterns of continuity in depression across the peripartum period. While strengths-based postpartum research is scarce, some evidence suggests that childbearing represents a positive life choice for a portion of young Black mothers (Geronimus, 2003; Oxford, Gilchrist, Gillmore, & Lohr, 2006) and can be associated with improved general functioning (Arai, 2009). Future studies examining positive postpartum adjustment and social determinants of well-being and flourishing across the transition to motherhood are clearly warranted.

### Limitations

The current study benefitted from extensive repeated measures data from a longitudinal cohort study with high participant retention, but several limitations should be considered alongside these strengths. The results were based on participants who are young, predominantly Black, first-time mothers living in a single urban region of the country. Sub-study exclusion criteria led to an over-representation in younger maternal age and Black race compared to the population-based parent study. In addition, these criteria may have biased the sample due to participants being screened out on post-exposure variables (e.g. preterm birth, mother in residential facility). Specifically, estimates may have been biased toward the null if continuities in depression were mediated by any of these factors, or away from the null if these factors were predictors of new-onset postpartum depression. Future work should explore the consistency of the results for multiparous women, and across diverse populations with respect to age, race, ethnicity, and geography. It is well-recognized that measurement continuity is a considerable challenge for understanding peripartum depression within a lifespan context. In the current study, preconception and prenatal depression symptom severity was assessed using a DSM-based measure to parallel assessment in clinical settings and allow for comparisons with non-childbearing samples, whereas depression in the postpartum period was assessed with the widely used EPDS that avoids the confounds of symptoms related to motherhood (e.g. loss of sleep, changes in weight). Nevertheless, results were consistent with prior research showing high levels of continuity from pregnancy to postpartum, although the true strength of this association may have been greater. Continuity via prenatal depression may also have been underestimated given that depression was only assessed on one occasion during pregnancy. Finally, although we assessed postpartum depression during the peak period prevalence of depression (Munk-Olsen et al., 2006), our findings may not reflect risk for women who develop depression later in the postpartum year.

## Conclusions

Understanding the impact of modifiable risks on depression in the postpartum period is imperative for developing appropriate intervention plans for improving health among women and their offspring. The current study is the first to demonstrate that, in addition to depression occurring during pregnancy, cumulative history of depression prior to conception is an important risk factor for depression in the postpartum period in a predominantly Black sample of young, first-time mothers. Given that women aged between 15 and 29 years are a high-risk group for depression onset and escalation (Lewis, Sae-Koew, Toumbourou, & Rowland, 2020; Shore, Toumbourou, Lewis, & Kremer, 2018) and up to 50% of school-age children experience emotional problems severe enough to warrant the use of mental health services (Kessler et al., 2012), there is already an urgent need for universal screening and mental health interventions across a variety of settings during this developmental period. Our findings further underscore this need, showing that the extended preconception period is a critical time, not only for identifying adolescents and young women who may be vulnerable following childbirth, but also for implementing preventive interventions that could disrupt developmental cascades and exacerbation of problems that could ultimately reduce postpartum depression-related impairment, morbidity, and mortality.

## Supplementary material

For supplementary material accompanying this paper visit https://doi.org/10.1017/S0033291722001210. click here to view supplementary material
