# Supplementary material for: A lifespan perspective on depression in the postpartum period in a racially and socioeconomically diverse sample of young mothers
Source: Psychol Med. 2022 May 6;53(10):4415–23. doi: 10.1017/S0033291722001210 (PMC9637236; doi:10.1017/S0033291722001210)

Figure 1
Illustration of cumulative depression over the 8 years pre-pregnancy for two women, both with a depression score during pregnancy of 7. Woman A (left) has a negative slope of depression scores with a decrease of 0.6 units per year and Woman B (right) has a positive slope with an increase of 0.2 per year.


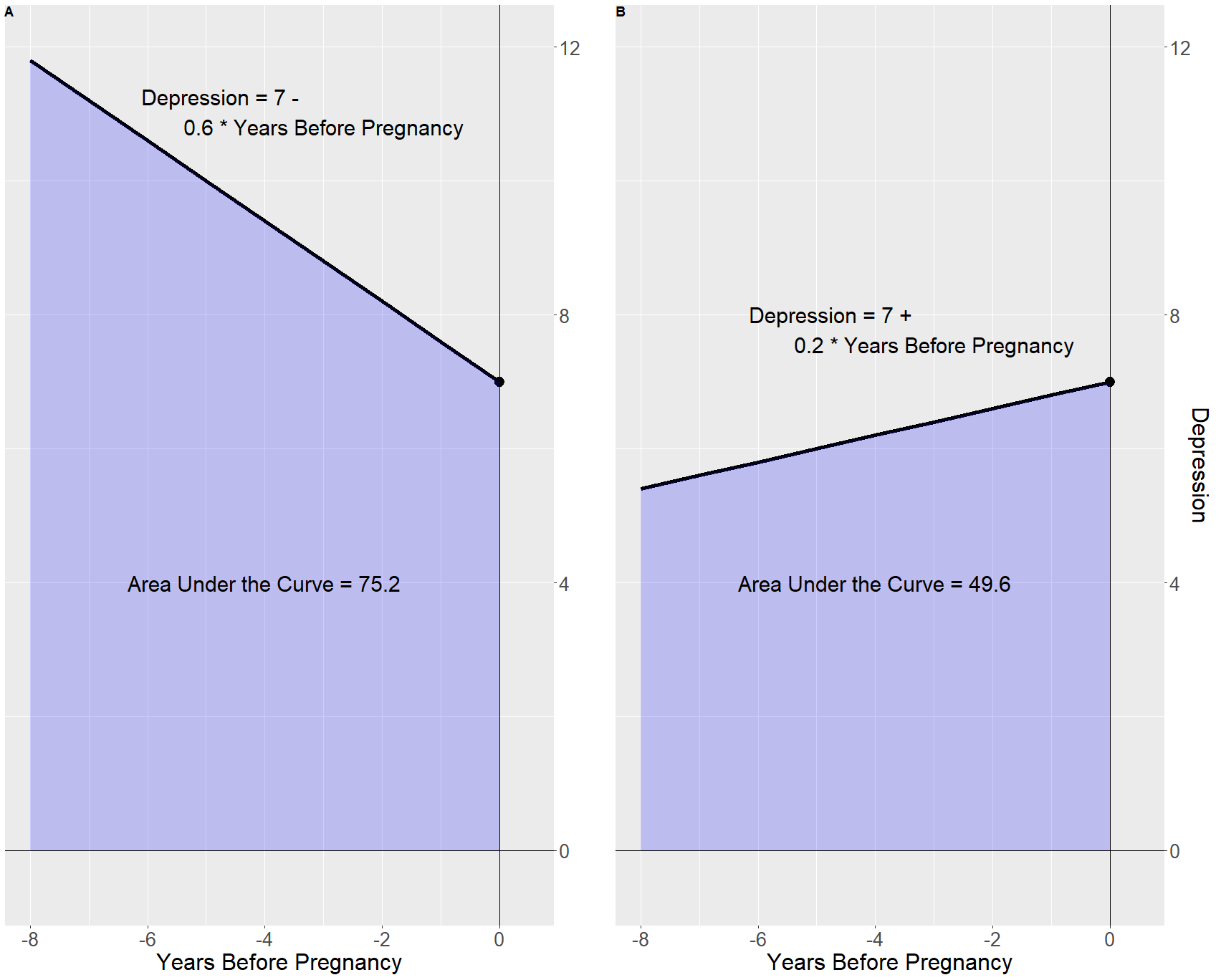


Figure 2

Illustration of the cumulative depression T-years pre-pregnancy for a woman with a slope in pre-pregnancy depression of “b”, and a pregnancy depression score of “a”.


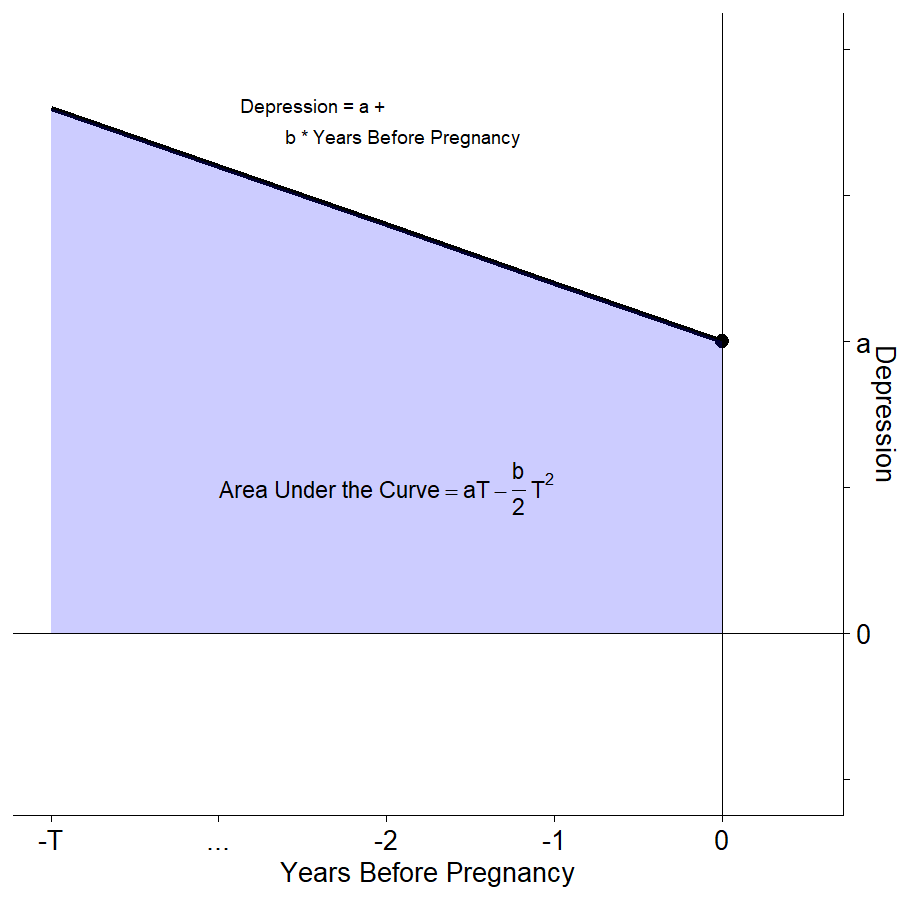

Supplement: Supplementary file 1 [file S0033291722001210sup.zip › S0033291722001210sup001.docx]
