# Supplementary material for: A lifespan perspective on depression in the postpartum period in a racially and socioeconomically diverse sample of young mothers
Source: Psychol Med. 2022 May 6;53(10):4415–23. doi: 10.1017/S0033291722001210 (PMC9637236; doi:10.1017/S0033291722001210)
Supplement: Supplementary file 1 [file S0033291722001210sup.zip › S0033291722001210sup002.docx]

Supplement to:
“A Lifespan Perspective on Depression in the Postpartum Period in a Racially and Socioeconomically Diverse Sample of Young Mothers”

Alison E. Hipwell^1,2^, Irene Tung^1,3^, Robert T. Krafty^4^, Audrey W. Leong^5^, Meredith Spada^1^,

Hope Vaccaro^6^, Sarah C. Homitsky^7^, Eydie Moses-Kolko^1^ and Kate Keenan^8^

^1^ Department of Psychiatry, University of Pittsburgh, USA

^2^ Department of Psychology, University of Pittsburgh, USA

^3^ Department of Psychology, California State University Dominguez Hills, USA

^4^ Department of Biostatistics and Bioinformatics, Emory University, USA

^5^ Department of Biological Sciences, University of Pittsburgh, USA

^6^ Department of Psychological Sciences, Case Western University, USA

^7^ Women’s Behavioral Health, Allegheny Health Network, Pittsburgh PA, USA

^8^ Department of Psychiatry and Behavioral Neuroscience, University of Chicago, USA

This supplement contains two sections: Section 1 provides details with regards to the interpretation of the slope of pre-pregnancy depression conditional on depression status during pregnancy, and its association with cumulative depression, and Section 2 provides results from a sensitivity analysis.

**Section 1: Interpretation of Pre-Pregnancy Slope Conditional on Depression During Pregnancy**

*Illustrative Example:* Before formally deriving the connection between the slope of pre-pregnancy depression and cumulative depression, we considering an example to illustrate this relationship. We consider data from two theoretical women. Both women have the same depression score of 7 during pregnancy. Woman A has a negative slope in pre-pregnancy depression of -0.6, or has a decrease in her depression score of 0.6 points per year leading up to pregnancy. Woman B has a positive slope in pre-pregnancy depression of 0.2, or has an increase in her depression score of 0.2 points per year leading up to pregnancy. Figure 1 displays the depression status for these two women over 8 years prior to pregnancy (the median pre-pregnancy observation time in our sample). Visually, it can be seen that the area under the depression curve, which measures the cumulative depression over the 8 years before pregnancy, is larger for the Woman A, who has a negative slope, compared to Woman B, who has a positive slope. Using the derivation that is presented in the subsequent section, we can find the area under the curve for Woman A is 75.2 over these 8 years, or an average depression score of 9.4 per year, while Woman B has an area under the curve of 49.6, or an average depression score of 6.2 per year. This helps illustrate that cumulative depression over a period of time pre-pregnancy as measured by the area under the depression trajectory is negatively proportional to the pre-pregnancy slope conditional on depression during pregnancy.

[INSERT FIGURE 1 HERE]

*Derivation:* The cumulative depression score defined as the area under the curve of the depression trajectory of a woman who has a slope of pre-pregnancy depression of “b” and a depression score during pregnancy of “a” over “T” years prior to pregnancy can be computed as

Area Under the Curve = $\int_{-T}^{0} \left( a+bt \right)dt=aT-\frac{b}{2}T^{2}$.

[INSERT FIGURE 2 HERE]

Conditional on a value of depression during pregnancy, the cumulative depression over a given time frame before pregnancy is inversely proportional to the slope of depression. For example, if a woman has a pregnancy depression score of 7 and a slope of “b”, then her cumulative depression over the 8 years prior to pregnancy is 56-32b. An increase in the slope by one unit is equivalent to a decrease in 8-year cumulative depression of 32.

**Section 2: Sensitivity Analyses**

To explore the potential effect of missing data on our inference, we refit the 3 regression models presented in the manuscript on two subsets of our data: once for participants who had no missing pre-pregnancy observations, and once for participants who have some missing pre-pregnancy observations.

Table 1. Regression models predicting postpartum depression scores from history of depression for the full sample, and separately for women with no missing depression scores and for women with missing depression scores.

|  | Combined Sample  B | Combined  Sample  95% CI | No  Missing  B | No  Missing  95% CI | With  Missing  B | With  Missing  95% CI |
| --- | --- | --- | --- | --- | --- | --- |
| *Model 1* |  |  |  |  |  |  |
| Age at delivery | -0.11 | (-0.33, 0.11) | -0.17 | (-0.40, 0.07) | 0.65 | (-0.09, 1.40) |
| Race^1^ | -1.79 | (-3.28, -0.31) | -2.39 | (-4.07, -0.70) | 1.01 | (-2.30, 4.31) |
| Prenatal public  assistance^2^ | -0.18 | (-1.39, 1.03) | -0.29 | (-1.59, 1.00) | 0.07 | (-3.41, 3.55) |
| Depression severity  during pregnancy | 0.39 | (0.27, 0.51) | 0.38 | (0.26, 0.51) | 0.42 | (0.07, 0.77) |
| *Model 2* |  |  |  |  |  |  |
| Age at delivery | -0.16 | (-0.41, 0.08) | -1.85 | (-3.68, -0.03) | 0.76 | (-0.01, 1.60) |
| Race^1^ | -1.54 | (-3.16, 0.08) | -0.10 | (-1.53, 1.32) | 0.76 | (-2.69, 4.22) |
| Prenatal public  assistance^2^ | 0.02 | (-1.31, 1.34) | 4.54 | (7.94, -0.57) | -0.57 | (-4.22, 3.07) |
| Slope of  Preconception  depression | 0.48 | (-0.50, 1.47) | 0.56 | (-0.43, 1.56) | 0.36 | (-0.13, 0.85) |
| *Model 3* |  |  |  |  |  |  |
| Age at delivery | -0.15 | (-0.37, 0.08) | -0.22 | (-0.45, 0.02) | -0.69 | (1.45, -0.06) |
| Race^1^ | -1.88 | (-3.35, -0.36) | -2.49 | (-4.18, -0.81) | -0.82 | (-4.16, 2.53) |
| Prenatal public  assistance^2^ | -0.32 | (-1.53, 1.34) | -0.52 | (-1.82, 0.79) | -0.05 | (-3.50, 3.60) |
| Depression severity  during pregnancy | 0.52 | (0.37, 0.68) | 0.55 | (0.38, 0.72) | 0.35 | (-0.07, 0.76) |
| Slope of  preconception  depression | -1.48 | (-2.57, -0.38) | -1.66 | (-2.85, -0.48) | -1.38 | (-6.59, 3.83) |

*Note*. ^1^Race coded as 1=Black American (includes multiracial Black and another race) vs. 0=White American. ^2^Public assistance coded as 0=no vs. 1=yes.
